# Dipeptidyl-Peptidase-IV Inhibitors, Imigliptin and Alogliptin, Improve Beta-Cell Function in Type 2 Diabetes

**DOI:** 10.3389/fendo.2021.694390

**Published:** 2021-09-20

**Authors:** Xu Liu, Yang Liu, Hongzhong Liu, Haiyan Li, Jianhong Yang, Pei Hu, Xinhua Xiao, Dongyang Liu

**Affiliations:** ^1^Savaid Medical School, University of Chinese Academy of Sciences, Beijing, China; ^2^Drug Clinical Trial Center, Peking University Third Hospital, Beijing, China; ^3^Clinical Pharmacology Research Center, Peking Union Medical College Hospital and Chinese Academy of Medical Sciences, Beijing, China; ^4^Department of Pharmacology, College of Pharmacy, Inner Mongolia Medical University, Hohhot, China; ^5^Center of Clinical Medical Research, Institute of Medical Innovation and Research, Peking University Third Hospital, Beijing, China; ^6^Department of Endocrinology, Key Laboratory of Endocrinology of National Health Commission, Peking Union Medical College Hospital and Chinese Academy of Medical Sciences, Beijing, China

**Keywords:** DPP-4 inhibitor, imigliptin, alogliptin, diabetes, oral minimal model

## Abstract

**Objects:**

Imigliptin is a novel dipeptidyl peptidase-4 inhibitor. In the present study, we aimed to evaluate the effects of imigliptin and alogliptin on insulin resistance and beta-cell function in Chinese patients with type-2 diabetes mellitus (T2DM).

**Methods:**

A total of 37 Chinese T2DM patients were randomized to receive 25 mg imigliptin, 50 mg imigliptin, placebo, and 25 mg alogliptin (positive drug) for 13 days. Oral glucose tolerance tests were conducted at baseline and on day 13, followed by the oral minimal model (OMM).

**Results:**

Imigliptin or alogliptin treatment, compared with their baseline or placebo, was associated with higher beta-cell function parameters (*φ*_s_ and *φ*_tot_) and lower glucose area under the curve (AUC) and postprandial glucose levels. The changes in the AUC for the glucose appearance rate between 0 and 120 min also showed a decrease in imigliptin or alogliptin groups. However, the insulin resistance parameter, fasting glucose, was not changed. For the homeostatic model assessment (HOMA-β and HOMA-IR) parameters or secretory units of islets in transplantation index (SUIT), no statistically significant changes were found both within treatments and between treatments.

**Conclusions:**

After 13 days of treatment, imigliptin and alogliptin could decrease glycemic levels by improving beta-cell function. By comparing OMM with HOMA or SUIT results, glucose stimulation might be more sensitive for detecting changes in beta-cell function.

## Introduction

Diabetes is a serious and global health concern. Today, there are 463 million patients with diabetes all over the world, and the number of patients with diabetes may increase up to 700 million by 2045 (1). Most of these patients are type 2 diabetes mellitus (T2DM). Although many antihyperglycemic agents are available on the market, more than a third of patients with diabetes cannot achieve or maintain an appropriate glycemic target (2). As a new class of anti-diabetic drugs, dipeptidyl peptidase-4 (DPP-4) inhibitors exhibit many favorable features, such as a low risk of hypoglycemia, weight neutrality, and a lower glycemic variability (3) with comparable glucose control capability (4). The American Diabetes Association recommends DPP-4 inhibitors as monotherapy or part of combination therapy with metformin for T2DM treatment. Imigliptin hydrochloride is a novel DPP-4 inhibitor chemically synthesized by Shandong Xuanzhu Pharma, Inc. (Shandong, China). Pre-clinical *in vitro* and *in vivo* studies have shown that imigliptin can specifically inhibit DPP-4 with a good tolerance (5, 6). The National Medical Products Administration has approved imigliptin for clinical trials, and it has finished single- and multiple-dose studies (unpublished data). Alogliptin has been approved in many countries worldwide. Besides its excellent anti-diabetic effect, alogliptin prevents the progression of carotid atherosclerosis and does not increase the risk of cardiovascular events (7–9). Therefore, alogliptin has been used as the positive drug in the multiple-dose study for imigliptin.

Insulin resistance and impaired beta-cell function are the two major pathophysiologic abnormalities of T2DM (10), and both involve glucose regulation. The complications and comorbidities (such as cardiovascular disease and renal impairment) are typically accompanied by progressive insulin resistance and impaired beta-cell function (2). The accurate quantification of insulin resistance and beta-cell function is very useful for (1) revealing the pathological features in patients, (2) suggesting a dosing regimen, and (3) estimating the effectiveness and safety based on the understanding of drug mechanism. The Pharmaceuticals and Medical Devices Agency (11) or the European Medicines Agency (12) recommends insulin sensitivity (SI) and beta-cell function assessments as efficacy evaluation for oral hypoglycemic agents or secondary endpoints for micro- and macrovascular complications.

Researchers have established many assessment methods for insulin resistance and beta-cell function, such as glucose clamp, intravenous glucose tolerance test (IVGTT), and oral glucose/mixed meal tolerance test. The clamp and IVGTT, with the application of the minimal model, are considered two golden standards for the assessment of insulin resistance and beta-cell function (13, 14). However, both assessments are complex for clinical application, considering the non-physiological state and high costs, and can cause some adverse effects, such as phlebitis. Beta-cell function or insulin resistance is also quantified using single data or area under the curve (AUC) values, such as fasting insulin level or insulinogenic index (15, 16). However, single data sets have a high variation and physiological differences, such as the fasting insulin level being the primary regulator of hepatic glucose production but not of muscle glucose production (17). For the AUC approach, disease or drug treatment may alter only a portion of the area or slope—for example, patients may experience delayed insulin secretion due to loss of first-phase insulin secretion while maintaining normal overall insulin production (17, 18). As an easy method, the homeostatic model assessment (HOMA) has been widely used for clinical and epidemiological research (19). The secretory units of islets in transplantation index (SUIT) is also a good clinical marker to evaluate the efficacy of anti-diabetic drugs on beta-cell function (20). It is worth pointing out that HOMA and SUIT are calculated based on fasting blood glucose, insulin, or C-peptide levels, which means that these only reflect the beta-cell function of patients under steady state but not under stimulated state (19). HOMA or SUIT is limited to assessing the response of beta-cells to dynamic blood glucose.

Compared with the above-mentioned two golden standard methods, oral glucose/mixed meal tolerance tests are more convenient and can reliably mimic the normal physiological state. The oral minimal model (OMM) is established on the shoulder of the minimal model and has been validated with clamp and minimal model (21–23). OMM is a powerful tool with simultaneous quantification of key glucose metabolism processes, including insulin action, secretion, and glucose rate of appearance (Ra), thus assessing the new drug effect on insulin resistance and beta-cell function, including both the dynamic and static states (24, 25). In the present study, we used OMM to evaluate the effects of imigliptin and alogliptin on insulin resistance and beta-cell function in Chinese T2DM patients. On the one hand, as we know, no study has assessed the insulin resistance and beta-cell function of alogliptin by OMM or minimal model. Our study would help clinicians better understand its underlying mechanism. On the other hand, we attempted to suggest dosage regimens of imigliptin through a comparison with alogliptin.

## Methods

### Data Source

Data from a randomized, double-blind, placebo- and positive-controlled study were used to assess the tolerability, pharmacokinetics, and pharmacodynamics of the repeated oral administration of imigliptin in T2DM (CTR20140677). Chinese subjects with naïve T2DM were recruited if they met the criteria as follows: newly diagnosed with T2DM, age between 18 and 65 years, body mass index of between 20 and 30 kg/m^2^ and body weight ≥50 kg, HbA1c between 6.5 and 11%, serum creatine less than the upper limit of normal, and hepatic transaminase level less than two times the upper limit of normal. The subjects were excluded from the study if they met one of the following criteria: patients with significant cardiovascular, hepatic, renal, gastrointestinal, immune, or neurologic system diseases; complications of diabetes, such as diabetic retinopathy; diastolic blood pressure >90 mm Hg, and systolic blood pressure >140 mm Hg; patients who received any anti-diabetic agents, diet pills, niacin, or glucocorticoid drugs within 1 month; patients who received some food that could affect CYP3A4, CYP2D6, and CYP2E1; patients with a history of alcohol abuse or drug abuse; patients showing electrocardiographic abnormality; hemorrhage or donation of more than 400 ml of blood within 8 weeks; or tumor.

In this multiple, ascending-dose study, 37 subjects with naïve T2DM were recruited and allocated to cohorts based on their order of entry into the study. There were three cohorts, including 25 mg imigliptin (low-dose group), 50 mg imigliptin (high-dose group), and 25 mg alogliptin (positive group). Within each cohort, the subjects were randomized to imigliptin or alogliptin and placebo (placebo group) once a day for 13 days. Following that, we have four groups: placebo (*n* = 6), 25 mg alogliptin (*n* = 9), 25 mg imigliptin (*n* = 11), and 50 mg imigliptin (*n* = 11). A total of 33 subjects finished the trial. All subjects tolerated imigliptin or alogliptin well. Each subject underwent two oral glucose tolerance tests (OGTT) (26) containing 75 g glucose on day -1 (baseline) and day 13. At the baseline, blood samples were collected at 0, 10, 20, 30, 60, 90, 120, 180, and 240 min for detection of plasma glucose levels and at 0, 30, 60, 120, 180, and 240 min for detection of insulin and C-peptide levels. **Table 1** shows that the baseline demographic characteristics of these patients were similar among the groups, except for body weight.

**Table 1:** Baseline characteristics of the volunteers.

	Placebo	Alogliptin, 25 mg	Imigliptin, 25 mg	Imigliptin, 50 mg
Number	5	8	10	10
Sex (male/female)	3/2	6/2	6/4	7/3
Age (year)	52.80 (6.69)	50.88 (4.94)	56.60 (8.47)	53.80 (6.11)
Weight (kg)	61.48 (7.86)	75.66 (7.61)*	77.64 (8.97)**	71.17 (7.41)
Height (cm)	159.80 (7.46)	166.50 (9.99)	167.30 (9.58)	165.10 (7.09)
Body mass index	24.05 (2.27)	27.47 (3.83)	27.70 (1.82)	26.07 (1.49)
FPG (mmol/L)	9.40 (2.33)	8.62 (1.29)	9.09 (2.64)	8.59 (1.56)
FSI (µU/ml)	5.13 (2.17)	8.55 (1.99)	9.25 (5.22)	8.27 (3.28)
FSC (ng/ml)	1.30 (0.34)	1.35 (0.17)	1.67 (0.69)	1.56 (0.64)
HbA1c%	7.94 (0.71)	6.88 (0.24)	7.72 (1.13)	7.66 (0.99)

Values are reported as mean (standard deviation).

FPG, fasting plasma glucose; FSI, fasting serum insulin; FSC, fasting serum C-peptide.

*P < 0.05, **P < 0.01, compared with placebo.

### Sensitivity Index and Beta-Cell Function Assessment

SI and beta-cell function, including *φ*_b_ (basal beta-cell function), *φ*_s_ (static index, the second-phase), and *φ*_tot_ (total beta-cell function index), were assessed using two separate OMM models, glucose minimal model and C-peptide minimal model, with SAAM II 2.3.1.1 software from the Epsilon Group. In the glucose minimal model, glucose effectiveness (SG), glucose absorption fraction (*F*), and apparent distribution volume of glucose (*V*) were fixed to population values (SG = 0.025 min^-1^, *F* = 0.9, and *V* = 1.45 dl/kg) (27). In the C-peptide minimal model, C-peptide kinetic parameters were derived from the anthropometric characteristics of the patients according to a previous study (28). Following the minimal model, parameters were constrained not to be less than zero. If the model identification returned a parameter value of zero, the identification was rejected. Identifications were also rejected if the parameter coefficients of variation were greater than 100% (29). Parameter *h* means that the threshold level for glucose concentration should be very close to the basal glucose value in the C-peptide model. If *h* was changed by more than 10% from the basal glucose level, a Bayesian approach prior or fixed to the basal glucose value would be used. Parameter *a* is the provision rate constant. When it was elevated, a Bayesian approach would be used. The subject was excluded if the parameter was unable to find a value. The disposition index (DI_tot_) was defined as *φ*_tot_ × SI (30). The model structure is illustrated in **Supplementary Table S1**. HOMA was used to assess insulin resistance by HOMA-IR and beta-cell function by HOMA-β. HOMA-IR and HOMA-β were calculated by R 3.6.2 and R Studio 1.1 software. HOMA-IR was defined as fasting glucose concentration (mmol/L) × fasting insulin concentration (μU/ml)/22.5. HOMA-β was defined as 20 × fasting insulin concentration (μU/ml)/[fasting glucose concentration (mmol/L) - 3.5]. SUIT was defined as 1,485 × fasting C‐peptide concentration (ng/ml)/[fasting glucose concentration (mg/dl)−61.8].

### Glucose Absorption Assessment

To investigate the potential effects of alogliptin and imigliptin on glucose absorption, the AUC of Ra in the first 120 min (AUC_Ra0-120_) was calculated, which was normalized by the total orally absorbed glucose (AUC_Ra0-240_) according to a previous study (24). AUC_Ra0-120_% was defined as AUC_Ra0-120/_AUC_Ra0-240_ × 100%.

### Statistical Analysis

Data were included in the OMM assessment if the OGTT was followed by the clinical trial protocol. Four patients who terminated the trial were excluded from the analysis.

For the primary outcomes of the OMM assessment, the model parameters were calculated using SAAM II 2.3.1.1 software and reported as median (25th–75th) percentiles. After weight correction, the two-way analysis of variance (two-way ANOVA) was used to assess the differences between treatments and visits for normally distributed and homogeneous variables. The *post-hoc* analysis was performed using the estimated marginal means test adjusted by Bonferroni. Otherwise, a Kruskal–Wallis test was used for the non-parametric test and Dunnett’s *t*-test adjusted by Bonferroni multiple comparisons. For the characteristics of the patient and the response to the glucose tolerance test, such as the change from baseline in fasting plasma glucose (△FPG), these were reported as median (25th–75th) percentiles. Comparisons were carried out using one-way ANOVA for normally distributed and homogeneous variables, followed by Tukey–Kramer’s test for *post-hoc* analysis. Otherwise, a non-parametric test was used as mentioned above.

Correlations of HOMA-IR and SI, HOMA-β, SUIT, and beta-cell function parameters were estimated using Pearson’s correlation test for normally distributed variables; otherwise, Spearman’s correlation test was adopted. All statistical analyses were performed by R 3.6.2 and R Studio 1.1 software.

## Results

### Plasma Glucose, Insulin, and C-Peptide Concentration–Time Curves

**Figure 1:**
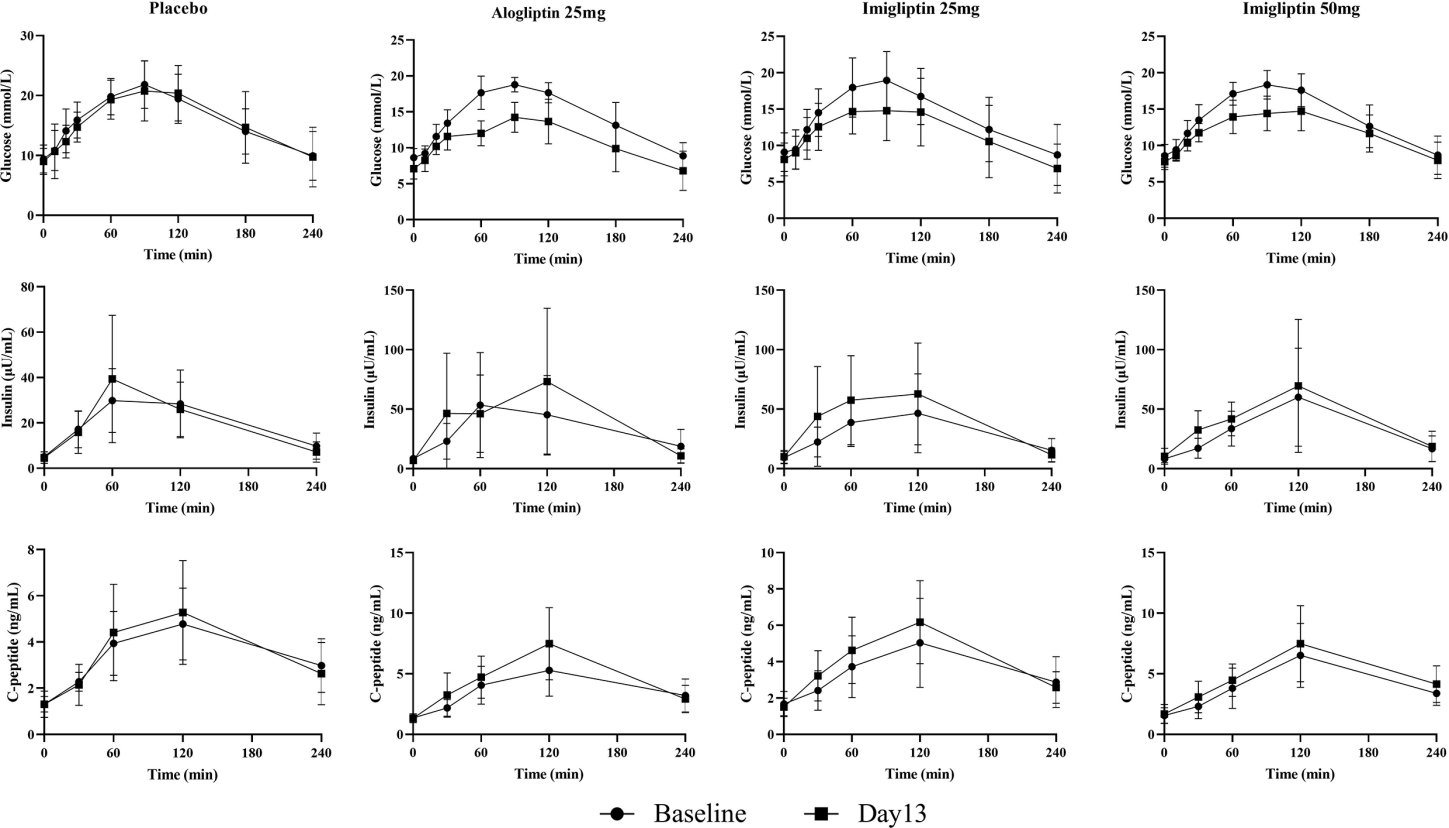
Glucose, insulin, and C-peptide concentration–time curves. Data are shown as mean ± SD.

**Figure 1** and **Table 2** show that imigliptin and alogliptin could produce a significant anti-diabetic effect compared with the placebo, exhibiting a decreased change from baseline in the AUC of glucose (△GAUC) and postprandial 2-h glucose (△PPG). After weight correction, no statistically significant changes were observed between imigliptin and alogliptin at △GAUC and △PPG. No statistically significant changes were observed in insulin or C-peptide between treatments, including the change from baseline in fasting serum insulin (△FSI) or fasting serum C-peptide (△FSC) and the change from baseline in the AUC of insulin (△IAUC) or C-peptide (△CAUC). However, it was worth pointing out that the medians of △FSI, △FSC, △IAUC, and △CAUC were increased.

**Table 2:** Glucose, insulin, and C-peptide outcomes.

Group	△FPG (mmol/L)	△PPG (mmol/L)	△GAUC (mmol/L × min)	△FSI (µU/ml)	△PSI (µU/ml)	△IAUC (µU/ml × min)	△FSC (ng/ml)	△PSC (ng/ml)	△CAUC (ng/ml × min)
**Placebo**	-0.50 (-1.10–0.50)	0.80 (-0.10–1.00)	16.50 (-283.50–314.50)	-0.55 [-0.79–(-0.01)]	1.65 (-6.06–4.01)	72.45 (-961.50–805.35)	0.00 (-0.20–0.10)	0.44 (-0.40–1.71)	21.90 (-34.80–179.85)
**Alogliptin, 25 mg**	-1.65 [-2.10–(-0.98)]	-3.80 [-5.22–(-2.67)]**	-836.00 [-1,010.62–(-523.62)]**	-1.43 (-2.90–0.09)	5.21 (0.63–32.13)	694.50 (-841.35–3,374.81)	0.10 (-0.25–0.22)	1.59 (0.90–2.67)	150.53 (54.83–385.12)
**Imigliptin, 25 mg**	-1.00 [-1.32–(-0.57)]	-1.85 [-3.30–(-1.02)]	-586.75 [-725.00–(-450.50)]*	-0.04 (-0.53–1.95)	9.99 (4.46–16.92)	1,522.72 (951.38–2,190.90)	-0.10 [-0.32–(-0.10)]	0.92 (0.76–1.29)	113.33 (59.21–203.14)
**Imigliptin, 50 mg**	-0.50 [-1.07–(-0.23)]	-2.50 [-2.77–(-2.00)]*	-415.75 [-654.75–(-325.62)]	1.59 (-1.19–5.52)	5.68 (1.00–16.67)	1,385.55 (882.41–2,214.19)	0.15 (-0.20–0.48)	1.23 (0.31–2.38)	206.85 (3.15–382.91)

Values are reported as medians (interquartile range).

△FPG, fasting plasma glucose change from baseline; △PPG, postprandial 2-h glucose change from baseline; △GAUC, area under glucose concentration–time curve change from baseline; △FSI, fasting serum insulin change from baseline; △PSI, postprandial 2-h serum insulin change from baseline; △IAUC, area under insulin concentration–time curve change from baseline; △FSC, fasting serum C-peptide change from baseline; △PSC, postprandial 2-h serum C-peptide change from baseline; △CAUC, area under C-peptide concentration–time curve change from baseline.

*P < 0.05, **P < 0.01, compared with placebo.

### Sensitivity Index and Beta-Cell Function Assessment

**Figure 2:**
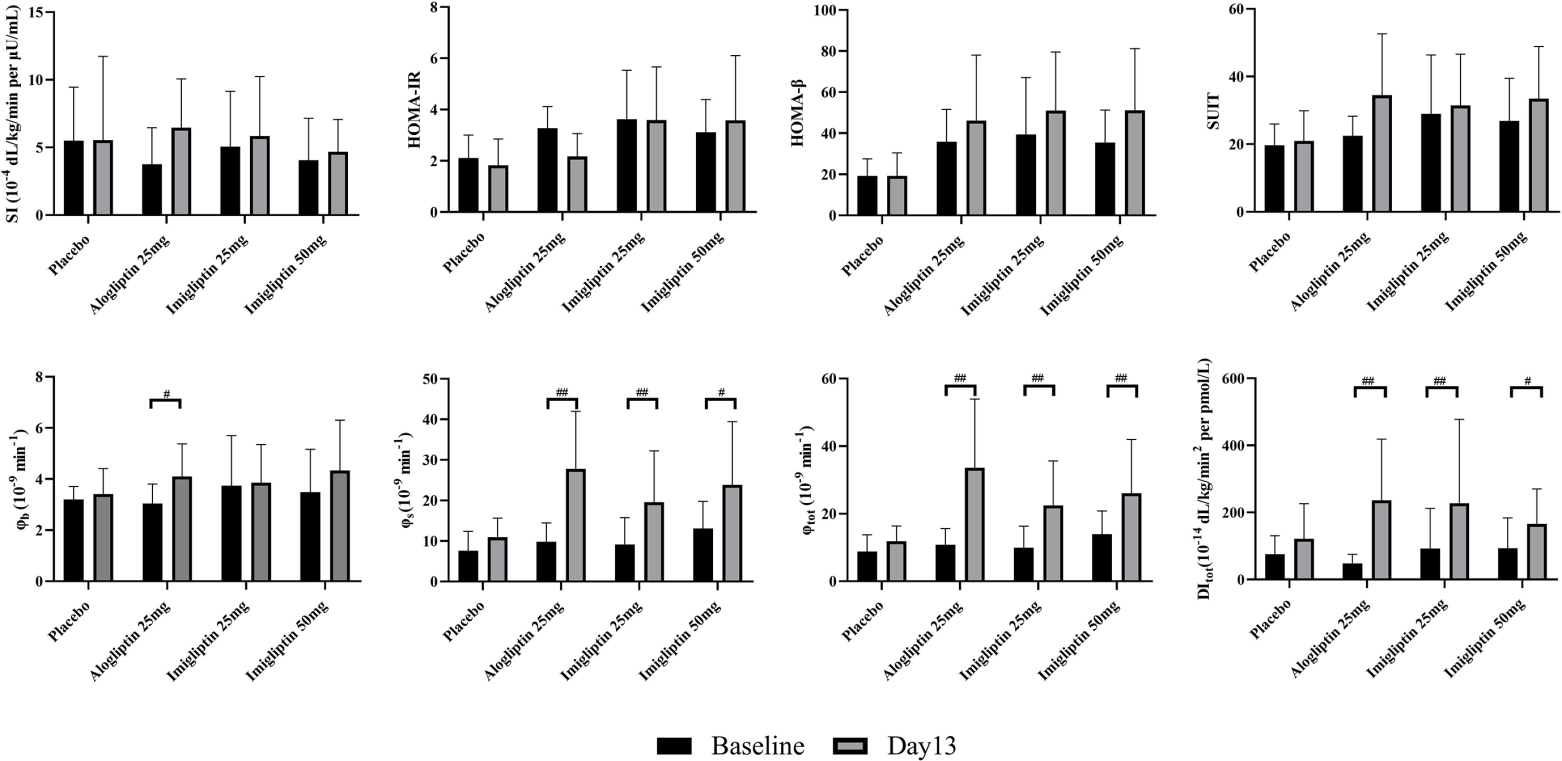
Insulin resistance and beta-cell function assessment by oral minimal model. Data are shown as mean ± SD. SI, insulin sensitivity; *φ*_b_, basal beta-cell function; *φ*_s_, static beta-cell function; *φ*_tot_, total beta-cell function; DI, total disposition index; HOMA-IR, insulin resistance by HOMA; HOMA-β, beta-cell function by HOMA; SUIT, secretory units of islets in transplantation index. ^#^ *P* < 0.05, ^##^ *P* < 0.01, day 1 compared with day 13.

**Supplementary Table S2** summarizes the parameter identification. For the OMM assessment, **Supplementary Table S3** (two-way analysis of SI and beta-cell function results) reveals that there was a statistically significant difference in *φ*_b,_ *φ*_s_, *φ*_tot_, and DI_tot_ by the visit after weight correction. No statistically significant differences were found in SI within groups or between groups. **Figure 2** shows that there were no differences in insulin resistance and beta-cell function parameters between groups at baseline or day 13. The *post-hoc* analysis showed that imigliptin or alogliptin had higher *φ*_s_ and *φ*_tot_ compared with their baseline values. However, it should be pointed out that the median values of the beta-cell function parameters (*φ*_s_ and *φ*_tot_) of imigliptin or alogliptin were increased by more than two times compared with placebo. No significant difference in beta-cell function was found between imigliptin and alogliptin. No changes in SI or beta-cell function parameters were observed in the placebo group.

For HOMA-β, HOMA-IR, and SUIT, a *post-hoc* analysis showed that no changes were observed within groups or between groups. The correlation coefficient of 0.81 and 0.96 indicated a strong positive correlation between HOMA-β or SUIT with *φ*_b_, but not *φ*_s_ or *φ*_tot_. HOMA-IR was poorly correlated with SI (*r* = -0.54).

### Glucose Absorption Assessment

**Figure 3:**
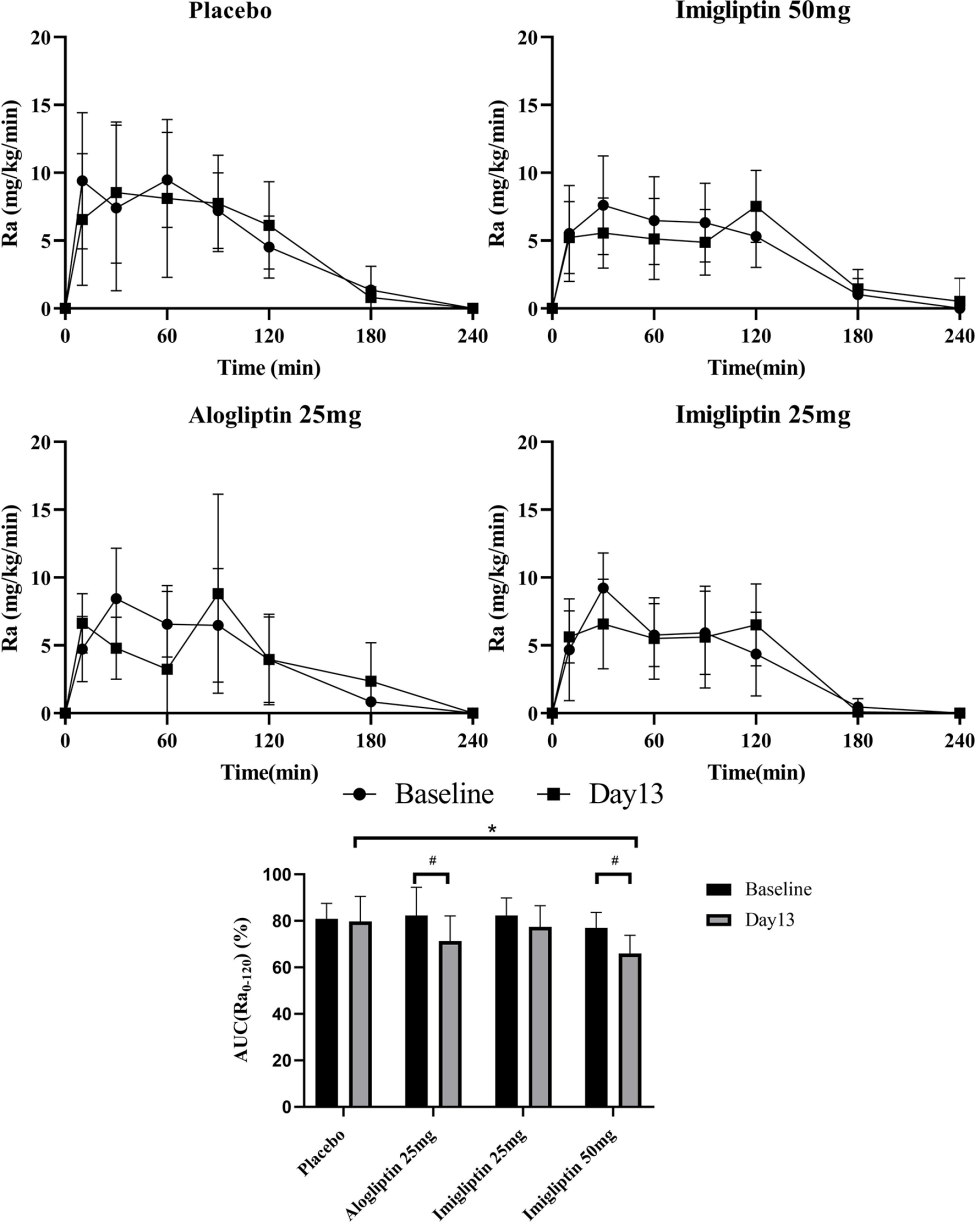
Glucose absorption rate–time curves and AUC(Ra_0-120_) %. Data are shown as means ± SD. AUC(Ra_0-120_), the area under the curve of Ra in the first 120 min. AUC(Ra_0-120_)% was defined as AUC(Ra_0-120_)/AUC(Ra_0-240_) × 100%. ^#^ *P* < 0.05, day 1 compared with day 13. *P < 0.05, compared with placebo.

**Figure 3** shows that the high-dose imigliptin and alogliptin decreased AUC_Ra0-120_% by about 11% compared with their baseline values. Compared with placebo on day 13, high-dose imigliptin showed a decrease in AUC_Ra0-120%_.

## Discussion

In the present study, imigliptin, the same as alogliptin, showed a good anti-diabetic effect in Chinese T2DM patients, exhibiting decreased △GAUC and △PPG (**Figure 1** and **Table 2**). Alogliptin showed an increase in beta-cell function parameters (*φ*_s_ and *φ*_tot_), but not SI (**Figure 2**). It was consistent with the previous studies and summarized in **Supplementary Table S4**. Vildagliptin or sitagliptin only improves beta-cell function parameters but does not change SI by OMM assessment (31). The glucagon-like peptide-1 (GLP-1) is an incretin hormone released in the intestine, and it can enhance insulin secretion but is rapidly degraded by DPP-4. Therefore, as a DPP-4 inhibitor, alogliptin can improve beta-cell function by increasing the GLP-1 levels in patients. Except for the increased incretin hormones, other potential mechanisms may include DPP-4 inhibitor-improved beta-cell survival, facilitated islet neogenesis, and anti-inflammatory effect (32).

Imigliptin had a similar result with alogliptin by the OMM assessment. No difference was found in beta-cell function or insulin resistance parameters between alogliptin and imigliptin, no matter if low or high dose. Considering the anti-diabetic effect, OMM assessment results, and safety, we suggested that 25 mg imigliptin was suitable for future study.

A meta-analysis study (32) has also shown similar results with a higher HOMA-β and unchangeable HOMA-IR after DPP-4 inhibitor treatment. SUIT has been demonstrated to be an effective technique for measuring beta-cell function in type 2 diabetes mellitus and is a valuable tool in the management of diabetic patients (20). It is very interesting to compare the OMM parameters and other assessment methods. No changes were observed in HOMA-IR, HOMA-β, or SUIT within groups or between groups. The correlation coefficient indicated that *φ*_b_ has a strong correlation with HOMA-β or SUIT, but not with *φ*_s_ or *φ*_tot_. HOMA-IR was poorly correlated with SI. The discrepancy might cause the difference between HOMA, SUIT, and OMM, as HOMA or SUIT parameters only considered basal plasma glucose, insulin, or C-peptide levels. It may take longer to observe a change in beta-cell function with drugs. Glucose stimulation might be more sensitive for probing the changes in beta-cell function.

Imigliptin or alogliptin showed a reduction in the glucose absorption rate during 120 min. L Ohlsson et al. (33) have found that glucose absorption is decreased after sitagliptin treatment. Moreover, GLP-1 could delay gastric emptying. A delayed or decreased glucose absorption might reduce the glucose absorption rate during 120 min. However, the glucose absorption fraction was fixed to 0.9. That means the OMM could only suggest a glucose absorption delay but not a decrease. In addition, Ra showed a spike at around 90–120 min at baseline in the placebo group and after treatment with imigliptin or alogliptin (**Figure 3**). The previous study confirmed the Ra curve fitted by oral minimum model in healthy subjects using a multi-tracer approach and discovered just one peak at around 30 min (27). The OGTT protocol for this study contained 25 sample time points during 240 min. However, a reduced OGTT protocol duration, such as seven samples over 120 min, showed a new peak at around 90 min (26). The limited number of samples were contributed to fit the Ra curve. For another reason, different groups have varying Ra curves—for example, the Ra curves of obese adolescents have more peaks (34). Additionally, tracer approaches have also been used to determine the Ra experimentally. It was found that most experimental data revealed a typical Ra pattern of an initial peak, followed by a second peak that gradually drops to zero. The peaks of the Ra curve may be determined by biphasic stomach emptying and small intestine transit time (35, 36). We also measured the plasma GLP-1 levels in OGTT. Both alogliptin and imigliptin could considerably raise the concentration of GLP-1 in the early phase of OGTT, but the GLP-1 concentration was rapidly lowered, and baseline concentrations were recovered after 120 min. The mean peak concentration was 26.24 and 38.04 pmol/L for alogliptin and imigliptin, which exceeded the concentrations of infused GLP-1 known to influence stomach emptying (37). The concentration of GLP-1 may cause a greater Ra in 90–120 min due to the reduction of stomach emptying rate and small bowel motility. However, some studies have shown that GLP-1 delays gastric emptying, whereas DPP-4 inhibitors do not (38, 39), which may cause the resulting rise in peripheral active GLP-1 concentrations to be not sustained, in marked contrast to concentrations observed during peripheral GLP-1 infusion. The reasons for the disparities in glucose absorption need to be researched in the future.

Some studies have suggested that DPP-4 inhibitors can improve SI by clamp (40–42). However, these patients have uncontrolled blood glucose and long-time observation of above 6 months. The improvement in SI may be related to the reduction in glucotoxicity or inflammatory factors (43). As the observation time was increased, imigliptin or alogliptin might also improve SI.

A previous meta-analysis has shown that insulin resistance and beta-cell function have ethnic differences when collecting data from clamp or IVGTT. East Asians have a better SI than Caucasians or Africans but with a worse beta-cell function (44). Three parameters (glucose effectiveness, apparent glucose volume of distribution, and glucose absorption fraction) are non-uniquely identifiable in OMM, so they are fixed to population values from the Caucasian data (45). Ethnic differences may affect OMM assessment. To investigate the ethnic differences in OMM parameters, we summarized the reported OMM parameters of T2DM from Caucasians, South Asians, Korean, Chinese, and Japanese in **Supplementary Table S5**. Unfortunately, we only included a few studies and could not conduct a meta-analysis. Although we did not draw a clear conclusion, these data could be used as a basis for ethnic differences in insulin resistance and beta-cell function study. The fixed parameters may affect the assessment accuracy of an individual. The validation study of OMM should be tested in Chinese patients.

In recent years, more studies have assessed insulin resistance and beta-cell function by OMM. R Huang has found that circulating retinol-binding protein 4 is associated with beta-cell function (46). R Visentin has used OMM to evaluate the treatment effect of dual glucagon-like peptide-1 receptor/glucagon receptor agonist SAR425899 (24). OMM is very useful, but it also has some defects, such as fixed parameters. Moreover, an individual calculation is undoubtedly very laborious for a research with a considerable sample size. Besides this, our study has some limitations. Only five time points were used to determine the insulin and C-peptide levels for each patient. However, samples at 10 and 20 min were essential for an accurate and precise estimation of dynamic beta-cell responsivity *φ*_d_. Therefore, we did not analyze *φ*_d_ in our study. The current analysis was carried out based on a phase-1 study with a modest sample size; larger studies are required to confirm the findings of improved beta-cell function.

Collectively, we showed that imigliptin, a new DPP-4 inhibitor, same as alogliptin, could exert an anti-diabetic effect by improving beta-cell function. Compared with HOMA or SUIT, the OMM parameters were more sensitive for probing the changes in beta-cell function. Besides this, imigliptin and alogliptin might cause delayed glucose absorption.

## Data Availability Statement

The original contributions presented in the study are included in the article/**Supplementary Material**. Further inquiries can be directed to the corresponding authors.

## Ethics Statement

The clinical trial was approved by the ethics committee of Peking Union Medical College Hospital and Chinese Academy of Medical Sciences. The patients/participants provided their written informed consent to participate in this study.

## Author Contributions

DYL, PH, and XXX designed the study and reviewed this manuscript. XL and YL analyzed the experiment data and model assessment. HZL was the doctor of the clinical trial. XL, JHY, and DYL prepared this manuscript. All authors contributed to the article and approved the submitted version.

## Funding

This work was supported by Key Clinical Projects of Peking University Third Hospital (grant number BYSY2018063).

## Conflict of Interest

The authors declare that the research was conducted in the absence of any commercial or financial relationships that could be construed as a potential conflict of interest.

## Publisher’s Note

All claims expressed in this article are solely those of the authors and do not necessarily represent those of their affiliated organizations, or those of the publisher, the editors and the reviewers. Any product that may be evaluated in this article, or claim that may be made by its manufacturer, is not guaranteed or endorsed by the publisher.
