# Supplementary material for: Dipeptidyl-Peptidase-IV Inhibitors, Imigliptin and Alogliptin, Improve Beta-Cell Function in Type 2 Diabetes
Source: Front Endocrinol (Lausanne). 2021 Sep 20;12:694390. doi: 10.3389/fendo.2021.694390 (PMC8488395; doi:10.3389/fendo.2021.694390)
Supplement: Supplementary file 1 [file DataSheet_1.docx]

**Supplement Table 1. Model structure of oral minimal model(1)**

The oral glucose minimal model was described by the following equations:

$$\dot{Q}\left( t \right)=-{[S}_{G}+X(t)]\times Q(t)+S_{G}\times Q_{b}+Ra(\boldsymbol{a}, t) Q(0)=Q_{b}$$

$$\dot{X}\left( t \right)=-p_{2}\times X\left( t \right)+p_{3}\times\left[ I\left( t \right)-I_{b} \right] X\left( 0 \right)=0$$

$$G\left( t \right)=\frac{Q(t)}{V}$$

$$SI=\frac{p_{3}}{p_{2}}\times V$$

$$Ra(t)=\left\{ \begin{aligned} a_{i-1}+\frac{a_{i}-a_{i-1}}{t_{i}-t_{i-1}}\times\left( t-t_{i-1} \right) for t_{i-1}\leq t\leq t_{i} \\ 0 otherwise \end{aligned} \right.$$

$$\int_{0}^{t} Ra\left( t \right)\cdot dt=\frac{D\times f}{BW}$$

Q is the amount of glucose in the accessible compartment, Q_b_: baseline of the amount of glucose ,G is plasma glucose concentration, I is plasma insulin concentration, I_b_ is basal values of plasma insulin concentration. X is insulin action, V is the distribution volume. S_G_, the fractional (i.e., per unit distribution volume) glucose effectiveness measuring glucose ability per se to promote glucose disposal and inhibit glucose production. p_2_ is the rate constant describing the dynamics of insulin action; p_3_ is the parameter governing the magnitude of insulin action.SI: insulin sensitivity index. Ra is described as a piecewise linear function with known break point t_i_ and unknown amplitude k_i.。_D is the total amount of ingested glucose. f is the absorption fraction. BW is the body wight.

The oral C-peptide minimal model was described by the following equations:

$$\dot{q}_{1}\left( t \right)=-\left( k_{01}+k_{21} \right)\times q_{1}\left( t \right)+k_{21}\times q_{2}\left( t \right)+ISR\left( t \right) q_{1}\left( 0 \right)=0$$

$$\dot{q}_{2}\left( t \right)=-\left( k_{12} \right)\times q_{2}\left( t \right)+k_{21}\times q_{1}\left( t \right) q_{2}\left( 0 \right)=0$$

$$ISR=y\left( t \right)+k_{G}\times\frac{dG(t)}{dt}$$

$$\dot{y}\left( t \right)=-\frac{1}{T} [ y\left( t \right)-\beta\times\left( G\left( t \right)-h \right)] y\left( 0 \right)=0$$

$$c_{1}(t)=\frac{q_{1}(t)}{V}$$

q_1_ and q_2_ are the C-peptide concentration in the accessible and peripheral compartments respectively. ISR is made up of two components: one proportional, through parameter k_G_, to glucose rate of change (dG/dt), and one representing insulin release that, after a delay T, occurs proportionally to plasma glucose level above a threshold, h, through parameter β.

| **Supplement Table 2. Clinical trial and model identification outcomes** | | | | | | | |
| --- | --- | --- | --- | --- | --- | --- | --- |
|  | Finish/identification | Unfinish/ un-identification | Statistical analysis | | | | |
|  |  |  | Number of patients | Placebo | Alogliptin 25mg | Imigliptin 25mg | Imigliptin 50mg |
| Clinical trial | 33 | 4 | 33 | 5 | 8 | 10 | 10 |
| Glucose minimal model | 33 | 0 | 32 | 5 | 8 | 9 | 10 |
| C-peptide minimal model | 31 | 2 | 31 | 4 | 7 | 10 | 10 |

| **Supplement table 3. Two-way analysis of variance for insulin sensitivity and beta-cell function** | | |
| --- | --- | --- |
| Parameters | Treatment F value (P-value) | Visit F value (P-value) |
| SI (10^-4^ dL/kg/min per μU/mL) | 1.08 (0.36) | 1.86 (0.18) |
| φ_b_ (10^-9^ min^-1^) | 0.35 (0.80) | 4.96 (0.03) |
| φ_s_ (10^-9^ min^-1^) | 2.27 (0.10) | 32.44 (<0.001) |
| φ_tot_ (10^-9^ min^-1^) | 1.81 (0.16) | 31.63 (<0.001) |
| DI_tot_ (10^-14^ dL/kg/min^2^ per pmol/L) | 0.28 (0.84) | 18.96 (<0.001) |
| HOMA-IR | 1.76 (0.17) | 1.35 (0.25) |
| HOMA-β | 1.60 (0.20) | 2.49 (0.12) |
| SUIT | 0.65 (0.59) | 2.25 (0.14) |
| AUC(Ra_0-120_)% | 4.65 (0.006) | 12.91 (<0.001) |
| SI: insulin sensitivity; φ_b_: basal beta cell function; φ_s_: statis beta cell function; φ_tot:_ total beta cell function. DI: total disposition index. HOMAIR: insulin resistance by homoeostasis model; HOMA-β: beta cell function by homoeostasis model. SUIT: secretory units of islets in transplantation index ; AUC (Ra_0-120_) (%): area under the rate of meal glucose appearance curve in the first 120 minutes normalized by the total orally absorbed glucose. | | |

| **Supplement Table 4. Oral minimal model application in dipeptidyl-peptidase-IV inhibitor development** | | | | | | | |
| --- | --- | --- | --- | --- | --- | --- | --- |
| Author | Year | Groups | Number and Disease | Glucose tolerance test | Beta cell function | Insulin resistance | Other Indexes |
| Brazg, R.(2) | 2007 | Sitagliptin 50mg BID + metformin or Placebo + metformin for 4 weeks | 28 T2DM | Oral meal | φ_s_,φ_tot↑_ | - | DI_s_, DI_tot_↑ |
| Dalla Man, C.(3) | 2009 | Vildagliptin 50mg QD for 10 days | 14 T2DM | Oral meal | φ_s_,φ_tot↑_ | - | DI_d_,DI_s_, DI_tot_↑ |
| Bock, G.(4) | 2010 | Sitagliptin 100mg QD or Placebo for 8 weeks | 22 Impaired fasting glucose | Oral meal | - | - | DI_s_, DI_tot_↑ |
| Williams-Herman, D.(5) | 2012 | Sitagliptin 50 mg + metformin 1000 mg BID, sitagliptin 50 mg + metformin 500 mg BID, metformin 1000 mg BID, metformin 500 mg BID, sitagliptin 100 mg QD or placebo for 24 weeks, Patients on placebo were switched in a blinded manner to metformin 1000 mg BID at week 24, then following by a 30-week treatment and an additional 50-week extension phase. | 1091 T2DM | Oral meal | φ_b_,φ_s_,φ_tot↑_ (24 weeks) | - | DI_tot_↑ (24 weeks) |

QD: once a daily

BID: twice a daily

T2DM: Type 2 Diabetes Mellitus

| **Supplement table 5. Ethnic differences of OMM parameters in patients with type 2 diabetes** | | | | | | | | | | | | | |
| --- | --- | --- | --- | --- | --- | --- | --- | --- | --- | --- | --- | --- | --- |
| Ethnic Group | Ethnic  description | N | OMM results | | | | |  | Characteristics of patients | | | | |
|  |  |  | SI (10^-5^ dL kg^-1^ min^-1^/pmol L^-1^ | φ_b_, (10^-9^ min-^1^) | φ_d_  (10^-9^) | φ_s_ (10^-9^ min^-1^) | φ_tot_ (10^-9^ min^-1^) |  | Age  (years) | Sex  (male/female) | BMI (kg m^-2^) | Fasting  plasma glucose  (mg/dL) | HbA1C % |
| Caucasian(6) | clear | 18 | 5.80±6.36 | 0.38±0.21 | 94.46±64.06 | 4.19±2.21 | 4.92±2.59 |  | 63.20 ± 7.60 | 9/9 | 29.30 ± 4.90 | - | - |
| South Asians(6) | clear | 23 | 7.60 ±6.71 | 0.42±0.10 | 114.52±175.05 | 8.33±5.95 | 9.43±7.58 |  | 52.30 ± 8.80 | 11/12 | 28.60 ± 4.10 | - | - |
| Korean(7) | clear | 14 | 13.30±11.80 | 2.90±1.20 | 170.90±138.70 | 13.90±6.50 | 15.50±7.20 |  | 53.80±9.40 | 9/5 | 24.50±3.50 | 146.20±27.60 | - |
| Japanese(8) | clear | 20 | 20.95±22.51 | 5.35±1.97 | 205.50±150.14 | 12.95±4.78 | 14.85±5.26 |  | 55.50±10.35 | 13/7 | 28.45±3.77 | 152.71 | 7.60±0.71 |
| Chinese | clear | 37 | 7.52±6.29 | 3.36±1.51 | 89.48±67.91 | 9.13±4.68 | 9.87±4.63 |  | 52.38±8.51 | 25/12 | 26.43±2.72 | 165.75±38.86 | 7.60±0.93 |
| Chinese*(9) | unclear | 27 | 1.84 [1.74-4.92] | - | 260.03 [144.96–349.31] | 23.29  [14.14–32.51] | 27.53  [14.81–38.61] |  | 45.82±6.23 | 16/11 | 25.47±2.19 | 118.00±27.74 | 6.43±1.18 |
| Caucasian(10) | unclear | 14 | 9.60±6.24 | - | 269.50±164.63 | 19.30±10.85 | 20.70±11.22 |  | 57.30±11.22 | - | 31.20±7.48 | 147.73±27.02 | 6.80±1.12 |
| Caucasian(3) | unclear | 14 | 6.96±6.15 | - | 621.00±688.46 | 24.50±15.71 | 28.90±19.46 |  | 53.10±7.48 | - | 33.90±5.61 | 142.33±33.69 | 6.10±0.75 |
| Data as Mean±SD, *median[interquartile range]  OMM：oral minimal model | | | | | | | | | | | | | |

**Reference:**

1. Cobelli C, Dalla Man C, Toffolo G, Basu R, Vella A, Rizza R. The oral minimal model method. *Diabetes* (2014) 63(4):1203-13. doi: 10.2337/db13-1198.

2. Brazg R, Xu L, Dalla Man C, Cobelli C, Thomas K, Stein PP. Effect of adding sitagliptin, a dipeptidyl peptidase-4 inhibitor, to metformin on 24-h glycaemic control and beta-cell function in patients with type 2 diabetes. *Diabetes, obesity & metabolism* (2007) 9(2):186-93. doi: 10.1111/j.1463-1326.2006.00691.x.

3. Dalla Man C, Bock G, Giesler PD, Serra DB, Ligueros Saylan M, Foley JE, et al. Dipeptidyl peptidase-4 inhibition by vildagliptin and the effect on insulin secretion and action in response to meal ingestion in type 2 diabetes. *Diabetes care* (2009) 32(1):14-8. doi: 10.2337/dc08-1512.

4. Bock G, Dalla Man C, Micheletto F, Basu R, Giesler PD, Laugen J, et al. The effect of DPP-4 inhibition with sitagliptin on incretin secretion and on fasting and postprandial glucose turnover in subjects with impaired fasting glucose. *Clin Endocrinol (Oxf)* (2010) 73(2):189-96. doi: 10.1111/j.1365-2265.2009.03764.x.

5. Williams-Herman D, Xu L, Teng R, Golm GT, Johnson J, Davies MJ, et al. Effect of initial combination therapy with sitagliptin and metformin on β-cell function in patients with type 2 diabetes. *Diabetes, obesity & metabolism* (2012) 14(1):67-76. doi: 10.1111/j.1463-1326.2011.01492.x.

6. Jainandunsing S, Wattimena JL, Rietveld T, van Miert JN, Sijbrands EJ, de Rooij FW. Post-glucose-load urinary C-peptide and glucose concentration obtained during OGTT do not affect oral minimal model-based plasma indices. *Endocrine* (2016) 52(2):253-62. doi: 10.1007/s12020-015-0765-9.

7. Lim MH, Oh TJ, Choi K, Lee JC, Cho YM, Kim S. Application of the Oral Minimal Model to Korean Subjects with Normal Glucose Tolerance and Type 2 Diabetes Mellitus. *Diabetes & metabolism journal* (2016) 40(4):308-17. doi: 10.4093/dmj.2016.40.4.308.

8. Tanaka K, Saisho Y, Manesso E, Tanaka M, Meguro S, Irie J, et al. Effects of Liraglutide Monotherapy on Beta Cell Function and Pancreatic Enzymes Compared with Metformin in Japanese Overweight/Obese Patients with Type 2 Diabetes Mellitus: A Subpopulation Analysis of the KIND-LM Randomized Trial. *Clinical drug investigation* (2015) 35(10):675-84. doi: 10.1007/s40261-015-0331-5.

9. Huang R, Yin S, Ye Y, Chen N, Luo S, Xia M, et al. Circulating Retinol-Binding Protein 4 Is Inversely Associated With Pancreatic β-Cell Function Across the Spectrum of Glycemia. *Diabetes care* (2020) 43(6):1258-65. doi: 10.2337/dc19-2432.

10. Basu A, Dalla Man C, Basu R, Toffolo G, Cobelli C, Rizza RA. Effects of type 2 diabetes on insulin secretion, insulin action, glucose effectiveness, and postprandial glucose metabolism. *Diabetes care* (2009) 32(5):866-72. doi: 10.2337/dc08-1826.
